# Atrio-ventricular mechanics and heart failure in Ebstein's anomaly - a cardiac magnetic resonance study

**DOI:** 10.1186/1532-429X-18-S1-O119

**Published:** 2016-01-27

**Authors:** Michael Steinmetz, Marike Broder, Johannes T Kowallick, Pablo Lamata, Shelby Kutty, Matthias Seehase, Christina Unterberg-Buchwald, Wieland Staab, Jan M Sohns, Gerd Hasenfuss, Thomas Paul, Joachim Lotz, Andreas Schuster

**Affiliations:** 1 Pediatric Cardiology and Intensive Care, Georg-August-University Goettingen Medical Center, Goettingen, Germany; 2 Cardiology and Pneumology, Georg-August-University Goettingen Medical Center, Goettingen, Germany; 3 Radiology, Georg-August-University Goettingen Medical Center, Goettingen, Germany; 4 Division of Imaging Sciences and Biomedical Engineering, The Rayne Institute, St. Thomas' Hospital, King's College London, London, UK; 5 Children's Hospital and Medical Center, University of Nebraska Medical Center, Omaha, NE USA

## Background

Ebstein's anomaly (EA) is a rare but clinically important congenital heart disease with potential affection of right ventricular(RV), right atrial (RA), left ventricular (LV) and left atrial (LA) function that may play a role in heart failure development. Thus, we sought to assess quantitative atrial and ventricular function in EA with CMR feature tracking, and to correlate changes in biatrial and biventricular performance with the severity of disease and clinical parameters of heart failure.

## Methods

Atrial and ventricular deformation parameters were calculated from myocardial feature tracking (2D CPA MR, TomTec Unterschleissheim, Germany) from 30 EA and 20 healthy control subjects at 1.5 Tesla. RA and LA performance was characterized using longitudinal strain and strain rate parameters quantifying reservoir function (total strain [Ells], peak positive SR [SRs]), conduit function (passive strain [Elle], peak early negative SR [SRe]) and booster pump function (active strain [Ella], late peak negative SR [SRa]). Ventricular performance was characterized using RV and LV global longitudinal strain (Ell) and LV circumferential and radial short axis strain (Ecc and Err). Additionally, volumetric measurements (QMass, Medis, Leiden, The Netherlands) for all cardiac chambers including the Total right/left volume-index and heart failure markers (BNP, NYHA class) were quantified.

## Results

RA reservoir and booster pump function were significantly impaired in the EA group as compared to controls using strain and volume metrics (see table 1) while conduit function was not different based on volumes. Changes in RA performance correlated significantly with markers of heart failure (NYHA, BNP and Total R/L-Volume Index). LA function in EA patients was also significantly impaired with atrial contractile function correlating with NYHA class. Furthermore, EA patients exhibited an impaired RV function (see table 1) also with a significant correlation with heart failure markers (RV Ell with NYHA class: r = 0.466, p = 0.012), whereas LV parameters only showed a non-significant trend towards reduced performance.

**Table 1:** Comparison of volumetric and functional parameters of EA patients and healthy controls.

	Patients	Controls	p-value	Patients	Controls	p-values
RIGHT ATRIUM				LEFT ATRIUM		
Reservoir function						
AEF total (%)	45.51 ± 11.66	55.91 ± 8.83	* 0.002	59.67 ± 10.03	66.23 ± 7.13	* 0.018
Ells (%)	19.73 ± 10.49	28.35 ± 11.31	* 0.009	17.08 ± 9.74	20.64 ± 5.77	* 0.017
SRs (/sec)	0.94 ± 0.37	1.19 ± 0.43	* 0.031	0.72 ± 0.32	0.89 ± 0.27	* 0.061
Conduit function						
AEF passive (%)	30.62 ± 10.38	32,82 ± 12.12	0.505	47.77 ± 12.43	49.10 ± 12.21	0.053
Elle (%)	13,93 ± 8.21	18.29 ± 8.85	0.068	12.72 ± 8.09	15.28 ± 5.30	* 0.025
SRe (/sec)	-0.65 ± 0.29	-0.90 ± 0.42	* 0.021	-0.76 ± 0.38	-1.08 ± 0.44	* 0.0007
Booster pump function						
AEF active (%)	14.89 ± 11.54	23.09 ± 11.66	* 0.020	11.89 ± 8.19	17.14 ± 8.95	0.143
Ella (%)	5.8 ± 4.89	10.06 ± 5.64	* 0.008	4.36 ± 3.33	5.36 ± 3.62	0.342
SRa (/sec)	-0.59 ± 0.40	-0.9 ± 0.48	* 0.020	-0.41 ± 0.32	-0.61 ± 0.35	* 0.047
RIGHT VENTRICLE				LEFT VENTRICLE		
EF total (%)	44.77 ± 8.33	52.99 ± 5.23	* < 0.001	59.07 ± 7.50	62.75 ± 7.67	0.281
Ell (%)	-13.48 ± 6.26	-19.66 ± 3.60	* < 0.001	-15.67 ± 4.96	-18.37 ± 4.78	0.067
Ecc (%)				-17.61 ± 4,63	-17.81 ± 2.31	0.87
Err (%)				25.65 ± 9.83	28.22 ± 9.75	0.378

P-value from t-test or Mann-Whitney-U-Test, *p = statistically significant = < 0.05. AEF: atrial ejection fraction; Ell: longitudinal strain; SR: strain rate; EF: ejection fraction; Ecc: circumferential strain; Err: radial strain

## Conclusions

Right atrial function is impaired in EA and can be quantified using CMR-FT. In combination with reduced quantitative RV longitudinal strain in the presence of preserved RV-EF, these quantitative changes may potentially represent early stages in heart failure development in EA. This is underpinned by a close correlation of decreased RA and RV deformational function with heart failure parameters such as NYHA, BNP and Total R/L-Volume Index. LA function is also impaired in EA, while LV function is still normal.

We suggest that in EA patients, CMR derived RA function and RV longitudinal strain may be used as more sensitive markers to detect early deterioration of right heart function. Moreover, LA deformation should be monitored for early detection of left heart dysfunction. Incorporating these measures into CMR assessment may help to improve clinical management of EA patients.

**Figure 1:**
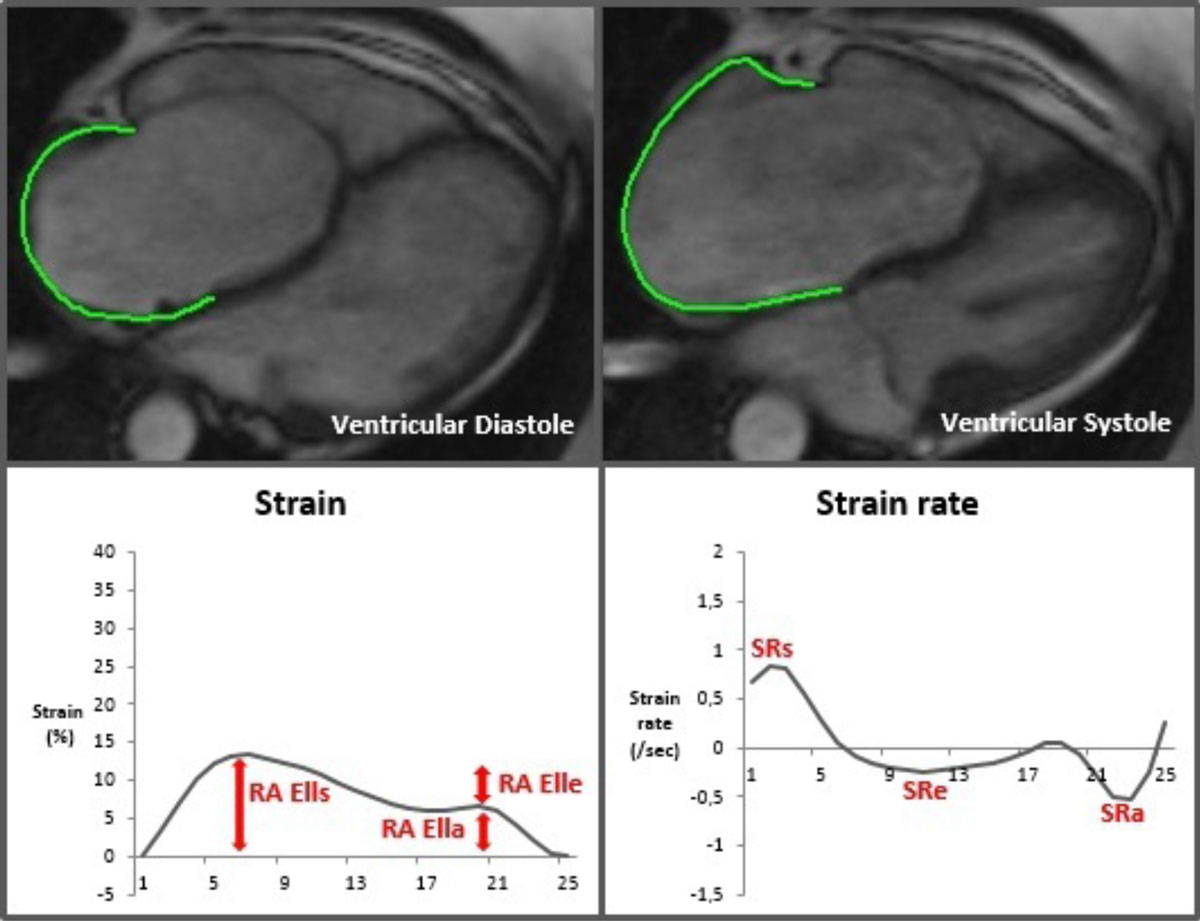
**Right atrial Feature Tracking in a patient with Ebstein's Anomaly**. RA Ell: right atrial longitudinal strain; SR: strain rate

